# CRISPRi links COVID-19 GWAS loci to LZTFL1 and RAVER1

**DOI:** 10.1016/j.ebiom.2021.103806

**Published:** 2022-01-06

**Authors:** Iris M. Fink-Baldauf, William D. Stuart, John J. Brewington, Minzhe Guo, Yutaka Maeda

**Affiliations:** a Perinatal Institute, Division of Neonatology, Perinatal and Pulmonary Biology, Cincinnati Children's Hospital Medical Center and Department of Pediatrics, University of Cincinnati College of Medicine (CCHMC and UC), Cincinnati, OH, USA; b Division of Pulmonary Medicine, Cincinnati Children's Hospital Medical Center and Department of Pediatrics, University of Cincinnati College of Medicine (CCHMC and UC), Cincinnati, OH, USA

**Keywords:** COVID-19, GWAS, CRISPRi, sn/scRNA-seq, Lung epithelial cells

## Abstract

**Background:**

To identify host genetic variants (SNPs) associated with COVID-19 disease severity, a number of genome-wide association studies (GWAS) have been conducted. Since most of the identified variants are located at non-coding regions, such variants are presumed to affect the expression of neighbouring genes, thereby influencing COVID-19 disease severity. However, it remains largely unknown which genes are influenced by such COVID-19 GWAS loci.

**Methods:**

CRISPRi (interference)-mediated gene expression analysis was performed to identify genes functionally regulated by COVID-19 GWAS loci by targeting regions near the loci (SNPs) in lung epithelial cell lines. The expression of CRISPRi-identified genes was investigated using COVID-19-contracted human and monkey lung single-nucleus/cell (sn/sc) RNA-seq datasets.

**Findings:**

CRISPRi analysis indicated that a region near rs11385942 at chromosome 3p21.31 (locus of highest significance with COVID-19 disease severity at intron 5 of *LZTFL1*) significantly affected the expression of *LZTFL1* (P<0.05), an airway cilia regulator. A region near rs74956615 at chromosome 19p13.2 (locus located at the 3’ untranslated exonic region of *RAVER1*), which is associated with critical illness in COVID-19, affected the expression of *RAVER1* (P<0.05), a coactivator of MDA5 (IFIH1), which induces antiviral response genes, including *ICAM1*. The sn/scRNA-seq datasets indicated that the MDA5/RAVER1-ICAM1 pathway was activated in lung epithelial cells of COVID-19-resistant monkeys but not those of COVID-19-succumbed humans.

**Interpretation:**

Patients with risk alleles of rs11385942 and rs74956615 may be susceptible to critical illness in COVID-19 in part through weakened airway viral clearance via LZTFL1-mediated ciliogenesis and diminished antiviral immune response via the MDA5/RAVER1 pathway, respectively.

**Funding:**

NIH.

**Research in context**

**Evidence before this study**

To identify genetic disparity in individual responses to COVID-19 disease severity other than sex, age and pre-existing health conditions, multiple GWAS have been conducted, which have led to the identification of several genomic loci that are involved in the severity of COVID-19 disease. However, most of the loci are in non-coding regions, which suggests that such loci influence the expression of nearby genes and in turn modulate critical illness in COVID-19. Genetic analyses including expression quantitative trait loci (eQTL) using non-specific cell types predict genes that are potentially affected by such loci; however, such analyses provide only correlative but not functional data.

**Added value of this study**

Recent advances in genome editing technology have led to the development of an epigenome editor, CRISPRi (interference), which can target specific loci in any genomic region, including non-coding regions. CRISPRi uses guide RNA (gRNA), which is designed to be specific to a locus, that recruits dCas9-KRAB to transcriptionally repress gene-regulatory activity of the locus. Thus, CRISPRi technology allows the identification of genes functionally affected by loci that were correlated to COVID-19 disease severity by GWAS. We further investigated the expression level of CRISPRi-identified genes at the single cell-level in human and monkey lungs infected with SARS-CoV-2 that causes COVID-19. Using CRISPRi and sn/scRNA-seq, we determined the potential functional roles of the loci that were identified by COVID-19 GWAS as being critical factors in COVID-19 disease severity.

**Implications of all the available evidence**

The data obtained from our CRISPRi analysis indicate that the expression of LZTFL1 (regulator of airway ciliogenesis) and RAVER1 (modulator of the MDA5 [also known as IFIH1] antiviral pathway) are functionally regulated by COVID-19 GWAS loci located at non-coding regions, respectively, suggesting that reduced airway viral clearance and a weakened antiviral immune response in lung epithelial cells are involved in the genetic disparity seen in COVID-19 disease severity. In fact, the sn/scRNA-seq data indicated that the MDA5/RAVER-ICAM1 pathway was activated in alveolar lung epithelial AT2 cells of mild-moderate COVID-19 cases in monkeys but not in those of severe COVID-19 cases in humans. Early detection of SARS-CoV-2 virus by frequent PCR testing and, if positive, early intervention by antibody-therapy targeting the virus may be beneficial for patients that carry SNPs rs11385942 and/or rs74956615 risk alleles. This therapeutic strategy would reduce the virus load to compensate for weakened airway virus clearance and impaired antiviral immune response in alveolar lung epithelial cells and could greatly improve the patients’ prognosis.

Alt-text: Unlabelled box

## Introduction

The recent novel coronavirus outbreak that occurred in Wuhan, China at the end of 2019 is caused by the SARS-CoV-2 coronavirus with the disease termed COVID-19. COVID-19 has become pandemic and is causing a global health and economic crisis.^1^ As of November 2021, COVID-19 is considered to be a respiratory infectious disease that causes severe pneumonia associated with symptoms such as cough, fever, fatigue, sputum production and/or shortness of breath resulting in death in many individuals.^2^, ^3^, ^4^ COVID-19 has caused more than 5 million deaths worldwide so far (https://covid19.who.int). However, most people with COVID-19 have mild or no symptoms.^5^ Factors such as age (>60), sex (male>female) and/or existing health conditions (e.g., respiratory/cardiovascular diseases and diabetes) have been reported to exacerbate the degree of symptoms and depress survival rate. However, there are severe cases where patients succumb to COVID-19 despite not having any of these underlying complicating factors.^3^^,^^6^^,^^7^ Human host genetic differences may account for the disparities in COVID-19 severity. To identify such genetic differences, a number of research projects using COVID-19 patients’ DNA and plasma/serum have begun worldwide mainly via genome-wide association studies (GWAS; e.g., the COVID-19 Host Genetics Initiative, https://www.covid19hg.org and the COVID Human Genetic Effort, https://www.covidhge.com), which led to identification of COVID-19-susceptible patients who carry risk variants in coding regions of type I IFN pathway genes (3.5% among the susceptible patients) and who develop autoantibodies against type I IFNs (10.2% among the susceptible patients),^8^^,^^9^ indicating lack of proper type I IFN responses exaggerates COVID-19 disease severity.

Among such projects, a study by the Severe Covid-19 GWAS Group indicated that SNPs, including rs11385942 (locus of highest significance at intron 5 of *LZTFL1*), at chromosome locus 3p21.21 are associated with the respiratory failure seen in COVID-19.^10^ Additional GWAS studies identified more loci associated with critical illness in COVID-19, including rs74956615 (at the 3’ untranslated exonic region of *RAVER1* at chromosome 19p13.2), rs2109069 (at intron 3 of *DPP9* at chromosome 19p13.3), rs6489867 (at intron 5 of *OAS1* at chromosome 12q24.13), rs10735079 (at intron 2 of *OAS3* at chromosome 12q24.13) and rs2236757 (at intron 6 of *IFNAR2* at chromosome 21q22.1).^11^^,^^12^ Since these SNPs are located at non-coding regions, including intronic and untranslated exonic regions, their loci are thought to influence pre-mRNA splicing/stability and/or affect gene expression if the regions harbouring the SNPs function as gene-regulators.

It used to be experimentally difficult to precisely determine beyond a correlative analysis (e.g., eQTL) the gene(s) whose mRNA expression level (single or multiple) is/are controlled by such non-coding regions, including intronic, intergenic, and untranslated exonic regions, that harbour disease-associated SNPs. However, the recent development of CRISPR/Cas9-mediated genome editing technology has enabled researchers to assess the gene-regulatory functions, if any, of such non-coding regions. Especially, an epigenome editor CRISPRi (a modified CRISPR/Cas9 in which dCas9-KRAB repressor transcriptionally represses the activity of gene-regulatory regions that are selected by sgRNA [single guide RNA]) has been shown to be useful to identify functional gene-regulatory regions at a genomic level using blood, kidney and prostate cell lines.^13^, ^14^, ^15^, ^16^, ^17^ We have recently adapted a CRISPRi approach using dCas9-KRAB expressing lung cell lines to streamline the functional assessment of non-coding regions that harbour lung disease-associated SNPs. Using this new CRISPRi approach, we previously identified genes regulated by such non-coding regions that harbour specific SNPs linked to chronic lung diseases, including asthma, cystic fibrosis (CF), chronic obstructive pulmonary disease (COPD) and idiopathic pulmonary fibrosis (IPF).^18^^,^^19^ In the present study using this same approach in lung epithelial cells, we sought to determine the functional roles of severe COVID-19 disease-associated SNPs as to whether they reside in gene-regulatory regions and which lung epithelial genes are affected by such regions. We further analysed the expression of affected genes at a single-cell level in human and monkey lungs infected with SARS-CoV-2 to identify a molecular pathway(s) involved in COVD-19 disease severity in lung epithelial cells *in vivo*.

## Methods

### CRISPRi (CRISPR interference) analysis

CRISPRi was performed as described previously^18^^,^^19^ except that gRNA (guide RNA) sequences were designed to target regions near SNPs that were significantly associated with COVID-19 disease severity independent of ATAC accessibility and the presence of histone gene-regulatory marks (H3K27ac, H3K4me1, H3K4me2, H3K4me3 and H3K9me3). Briefly, gRNA target sequences were designed using CRISPOR^20^ and synthetic sgRNAs (single gRNA) were generated using the Invitrogen custom TrueGuide gRNA ordering tool (Thermo Fisher, Waltham, MA). Non-targeted synthetic sgRNA was used as a negative control (cat#A35526, Thermo Fisher). A lentiviral vector harbouring CRISPR/dCas9-KRAB was obtained from Addgene (pLV hU6-sgRNA hUbC-dCas9-KRAB-T2a-Puro; Plasmid #71236).^21^ A lentiviral vector that carries dCas9-KRAB and sgRNA that targets a region near rs11385942 was made using DNA oligos obtained from Integrated DNA Technologies (Coralville, IA). Lentivirus was produced using the lentiviral vectors at the Viral Vector Core at Cincinnati Children's Hospital Medical Center (CCHMC). A549 and H1793 lung epithelial (carcinoma) cell lines that were obtained from ATCC (Manassas, VA), identification of which were validated by Laboratory Corporation of America Holdings (Burlington, NC), were used to generate cell lines that stably express dCas9-KRAB alone or dCas9-KRAB+sgRNA using the lentiviruses described above by puromycin selection (10 ug/ml at the final concentration). The absence of mycoplasma was confirmed using Universal Mycoplasma Detection Kit (cat#30-1012K, ATCC). The cell lines that express dCas9-KRAB were transfected with synthetic sgRNAs (50 nM final concentration) using Lipofectamine RNAiMAX Reagent (cat#13778075, Thermo Fisher). Two days after transfection, RNA was extracted using TRIzol reagent (cat#15596018, Thermo Fisher) and cDNA was made from the RNA using iScript^TM^ Reverse Transcription Supermix for Real-Time (RT)-qPCR (cat#1708841, Bio-Rad, Hercules, CA). TaqMan RT-qPCR gene expression analysis (Thermo Fisher) was performed using the cDNA to assess mRNA expression according to the manufacturer's protocol using probes (Hs01890924 for *CCR9*, Hs00373589 for *DPP9*, Hs00365407 for *FDX2*, Hs01089321 for *FYCO1*, Hs02758991 for *GAPDH*, Hs00913466 for *ICAM3*, Hs00175123 for *IL10RB*, Hs01066116 for *IFNAR1*, Hs01022060 for *IFNAR2*, Hs01118910 for *LARS2*, Hs00170931 for *LIMD1*, Hs00947898 for *LZTFL1*, Hs00211043 for *MRPL4*, Hs00384077 for *MYDGF*, Hs00973637 for *OAS1*, Hs00942643 for *OAS2*, Hs00196324 for *OAS3*, Hs00913477 for *RAVER1*, Hs01080325 for *SACM1L*, Hs00610960 for *SLC6A20*, Hs00177464 for *TYK2*, Hs00913460 for *ZGLP1*). A probe for dCas9 was custom-designed by Thermo Fisher (F-CCTGGATTTTCTTAAGTCCGATGGA; R-GAGAGTCATCATGGATCAACTGCAT; probe-CCAACCGGAACTTC).

### Visualization of chromatin modifications

ENCODE DNase-seq, ATAC-seq, and ChIP-seq signals and peaks on A549 cell lines^22^ were visualized using the UCSC Genome Browser.^23^ Data were downloaded from Gene Expression Omnibus (GEO) for H3K27ac (GSM2421872), H3K4me1 (GSM2421768), H3K4me2 (GSM2421626), H3K4me3 (GSM2421502), H3K9me3 (GSM2421866) and ATAC-seq (GSM3137776). DNase-seq signals (ENCODE ID: ENCFF541BTE) and peaks (ENCODE ID: ENCFF450PLG) were downloaded from the ENCODE web portal (https://www.encodeproject.org/experiments/ENCSR000ELW/).

### Single-nucleus RNA-seq analysis of human lungs infected with SARS-CoV-2

Processed data (UMI matrix and cell metadata) of single nucleus RNA-seq (snRNA-seq) of normal human lung were downloaded from GSE161383 (https://www.ncbi.nlm.nih.gov/geo/). Data from the three adult lungs (donor number: D122, D175 and D231, ages 29-33 years) were used for downstream analysis.^24^ For snRNA-seq analysis of COVID-19 human lung,^25^ raw UMI matrices were downloaded from GSE171668 (https://www.ncbi.nlm.nih.gov/geo/). The following 5 COVID-19 donors with ages between 30 and 60 (patient number: D3, D4, D10, D12, D15) were included in our analysis. Cell selection and associated cell type annotations were obtained from the original study.^24^^,^^25^ For normal human lung data, the “celltype” annotation was used^24^; 473 cells annotated as “Unclassified” were excluded. For COVID-19 human lung data, the “predicted_celltype” annotation was used^25^; 271 cells without predicted annotation were excluded. In total, the snRNA-seq of 9,920 cells from 3 normal lungs and 27,792 cells from 5 COVID-19 lungs were used for downstream analysis. To increase the number of common cell types in the normal and COVID-19 cell type assignments, some cell sub-types were merged as follows: for the normal lung data, vascular endothelial cell subtypes (Cap1, Cap2, veins and arteries), fibroblast cell subtypes (matrix fibroblast 1 and 2) and macrophage cell subtypes (alveolar and interstitial macrophages) were merged; for the COVID-19 data, monocyte cell subsets (“CD16+ monocyte” and “monocyte”) and T cell subsets (“CD4+ T cell, “CD8+ T cell” and “Treg”) were merged. For the additional validation study, processed data (UMI matrix and cell metadata) of snRNA-seq of COVID-19 (n=19) and control (n=7) human lungs (age 58-84) of Columbia cohort^26^ were downloaded from GSE171524 (https://www.ncbi.nlm.nih.gov/geo/). The “cell_type_fine” annotation from the original study was used as cell type annotation. Seurat (version 4.0.2, https://satijalab.org/seurat/) was used for single cell data management, normalization and Dotplot visualization.^27^ Differential expression between COVID-19 vs. normal cells of each common cell type was performed using the default differential expression pipeline in the Seurat 4 FindMarkers function, which conducts Wilcoxon rank sum test on data normalized using the “LogNormalize” method, a total count normalization that accounts for library size difference in individual cells.

### Single-cell RNA-seq analysis of African green monkey lungs infected with SARS-CoV-2

Single-cell RNA-seq (scRNA-seq) of African green monkey lungs (n=10, Animal IDs: AMG1-10) infected with SARS-CoV-2 via a combination of intranasal (0.5 ml per nostril), intratracheal (4 ml), oral (1 ml) and ocular (0.25 ml per eye) administration of a 4 × 10^5^ TCID_50_/ml (3 × 10^8^ genome copies/ml) virus dilution in sterile Dulbecco's modified Eagle's medium (DMEM)^28^ were downloaded from GSE156755 (https://www.ncbi.nlm.nih.gov/geo/). To remove low quality and potential doublet cells, the following quality control (QC) criteria were applied to cell prefiltering, including passing cell ranger QC, at least 500 but less than 5000 expressed genes, less than 5% of UMIs mapped to mitochondrial genes and less than 30000 total UMIs. The QC criteria were selected based on inspection of distributions of each QC metric. In total, 43,689 cells were included in downstream analysis using Seurat (version 4.0.2).^27^ Data were normalized using SCTransform function in Seurat and regressed out the effects of cell cycle and mitochondrial percentage. The SCTransform normalized data was used for principal component analysis to perform dimension reduction and identify principal components representing major sources of variation in the data. Top 200 principal components were selected based on the inspection of scree plot and were used for Harmony^29^ analysis to integrate data from different lungs. Unbiased cell clustering was performed using the Leiden algorithm.^30^ Cell clusters were mapped to cell types based on expression of known cell type marker genes from the LungMAP studies.^24^^,^^31^ Differential expression analysis was performed using the Seurat “LogNormalize” data with Wilcoxon rank sum test implemented with the FindMarkers function in Seurat, as described above. P-values were adjusted using Bonferroni correction for multiple testing among the three groups. Kruskal-Wallis rank sum tests were also conducted to compare all three groups.

### Ethics statement

All human (GSE161383, GSE171668 and GSE171524) and monkey (GSE156755) datasets were obtained from Gene Expression Omnibus (https://www.ncbi.nlm.nih.gov/geo/) and are compliant with human and animal ethics considerations.

### Statistics

RT-qPCR results were normalized against GAPDH in independent biological triplicates. Results are expressed as the mean ± SD of the triplicates for each group. Statistical relevance was determined using two-sided Student's *t*-test on targeted sgRNA-transfected vs. non-targeted sgRNA-transfected relative expression except that ratio paired *t*-test was used for Supplementary Fig. S4b. P values (P<0.05) obtained by two-sided Student's *t*-test that pass the Shapiro-Wilk normality test are reported for 1.5 or more-fold change in endogenous genes. GraphPad Prism 9 was used for graphing and statistical analysis. Statistical significance of single-nucleus/cell RNA-seq analyses were obtained using two-tailed Wilcoxon rank sum test. P values were adjusted using Bonferroni correction for multiple testing among the three groups in scRNA-seq of African green monkey lungs. Kruskal-Wallis rank sum tests were also conducted on three-group comparisons.

### Role of funders

Funders had no role in interpreting the data and publishing the study.

## Results

### A locus with SNP rs11385942 affects the expression of *LZTFL1*

**Figure 1:**
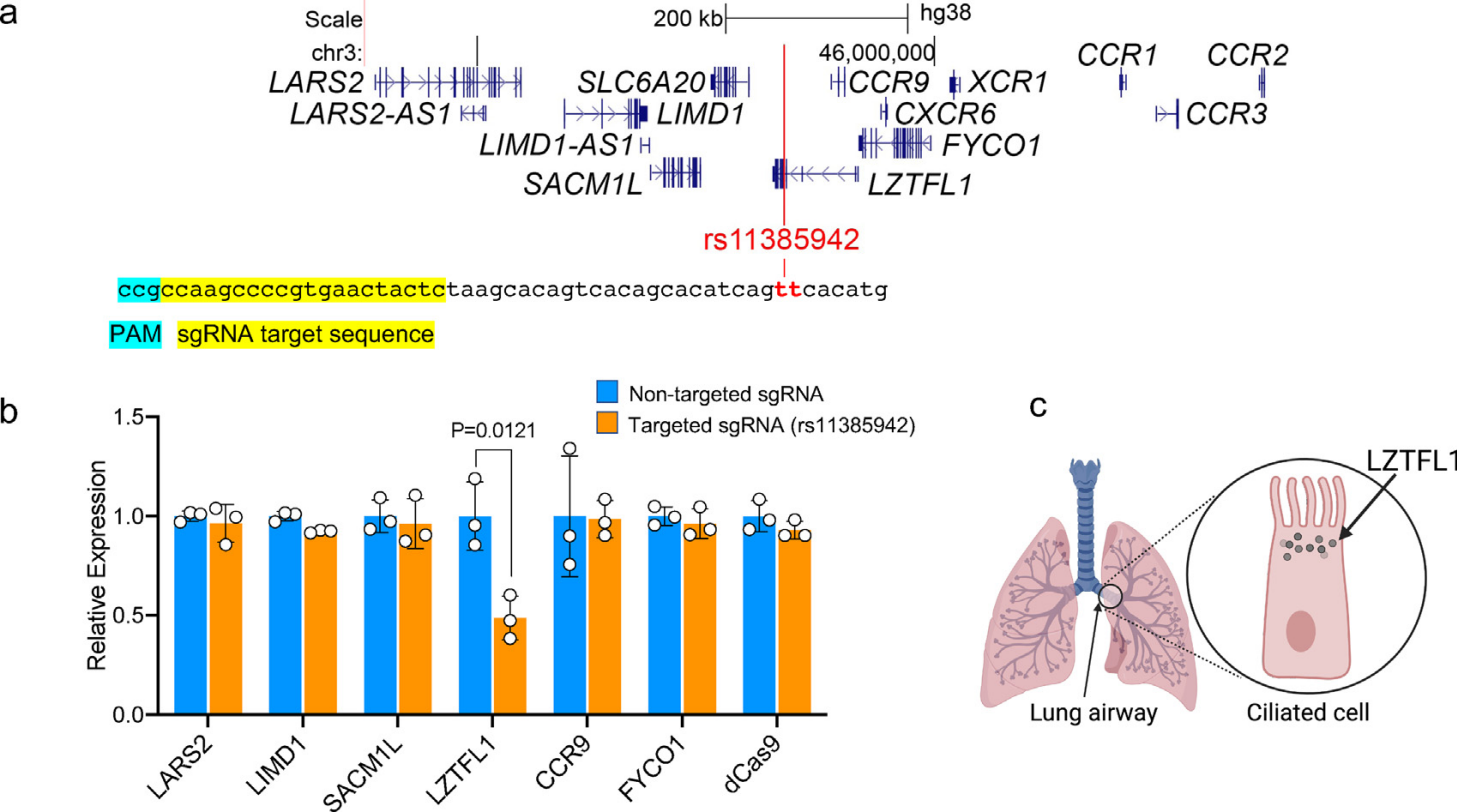
**A non-coding region located near SNP rs11385942 that is linked to COVID-19 disease severity regulates the expression of *LZTFL1*.** (a) SNP rs11385942 (red), most significantly associated with severe COVID-19 respiratory failure, is located at intron 5 of *LZTFL1* on chromosome 3p21.31. The guide RNA (gRNA) target sequence (yellow highlighted) along with PAM sequence (blue highlighted) near rs11385942 that was used for subsequent CRISPRi analysis is shown. The intronic sequence is in lowercase. (b) CRISPRi analysis was conducted using synthetic single gRNA (sgRNA) targeting the intronic region near rs11385942 in an A549 lung epithelial cell line that stably expresses dCas9-KRAB. The analysis indicates that the sgRNA repressed the expression of *LZTFL1*, but no other expressed lung epithelial genes located near the 3p21.31 locus compared to non-targeted control sgRNA. Three independent experiments were conducted. Error bars are ± SD. P values (two-sided Student's *t*-test) are reported for 1.5 or more-fold change in endogenous genes. (c) Lung diagram showing location of ciliated cells in the airways and LZTFL1 expression promoting ciliogenesis. The figure drawings were created with BioRender.com.

To determine whether loci that were identified by GWAS as linked to COVID-19 disease severity function as gene-regulatory regions, we used a CRISPRi approach focused on lung epithelial cells. Lung epithelial cells are critical cell types for COVID-19 pathogenesis.^32^ Therefore, we used lung epithelial cell lines that stably express dCas9-KRAB repressor that we previously created.^18^ We first analysed a locus harbouring rs11385942 to determine whether the locus functions as a gene-regulatory region since this locus has the highest significance linked with COVID-19 disease severity^10^. CRISPRi can repress the transcriptional activity of a genomic region by targeting the corresponding region with sgRNA (single guide RNA) along with a dCas9-KRAB repressor.^21^ Using the CRISPOR tool,^20^ we found a highly specific and efficient sgRNA target sequence (yellow highlighted) near rs11385942 at intron 5 of *LZTFL1* at chromosome locus 3p21.21 (Fig. 1a and Supplementary Fig. S1a). As previously performed,^18^ we transfected synthetic sgRNA targeting the locus in dCas9-KRAB-expressing A549 lung epithelial cells and conducted gene expression analysis assessing the expression of nearby genes, including *LARS2, LIMD1, SACM1L, LZTFL1, CCR9* and *FYCO1*, located at chromosome locus 3p21.21. Notably, the expression of *LZTFL1* but no other nearby lung epithelial genes was significantly repressed by the targeted sgRNA (≥1.5-fold, P=0.0121, two-sided Student's *t*-test; Fig. 1b). To validate the result obtained using synthetic sgRNA, we also produced lentivirus carrying both target sgRNA and dCas9-KRAB and infected A549 cells with the lentivirus and assessed the expression of the genes mentioned above. The results using A549 cells with the lentivirus were consistent with the results using synthetic sgRNA (≥1.5-fold, P=0.0036, two-sided Student's *t*-test; Supplementary Fig. S1b). Since A549 cells do not express endogenous *SLC6A20*, we repeated the same CRISPRi experiment using H1793 lung epithelial cells that express endogenous *SLC6A20*,^33^ which indicated that the expression of only *LZTFL1* but no other nearby lung epithelial genes, including *SLC6A20*, was significantly affected by the sgRNA targeting the locus (≥1.5-fold, P=0.0041, two-sided Student's *t*-test; Supplementary Fig. S1c). These results indicate that the locus with rs11385942 affects the expression of *LZTFL1* and may in turn influence COVID-19 severity. LZTFL1 regulates airway ciliogenesis (Fig. 1c),^34^ which is critical for virus clearance from airways, suggesting insufficient SARS-CoV-2 virus clearance in airways with patients carrying rs11385942 risk allele may lead to the progression of COVID-19 disease severity.

### A locus with SNP rs74956615 affects the expression of *RAVER1*

**Figure 2:**
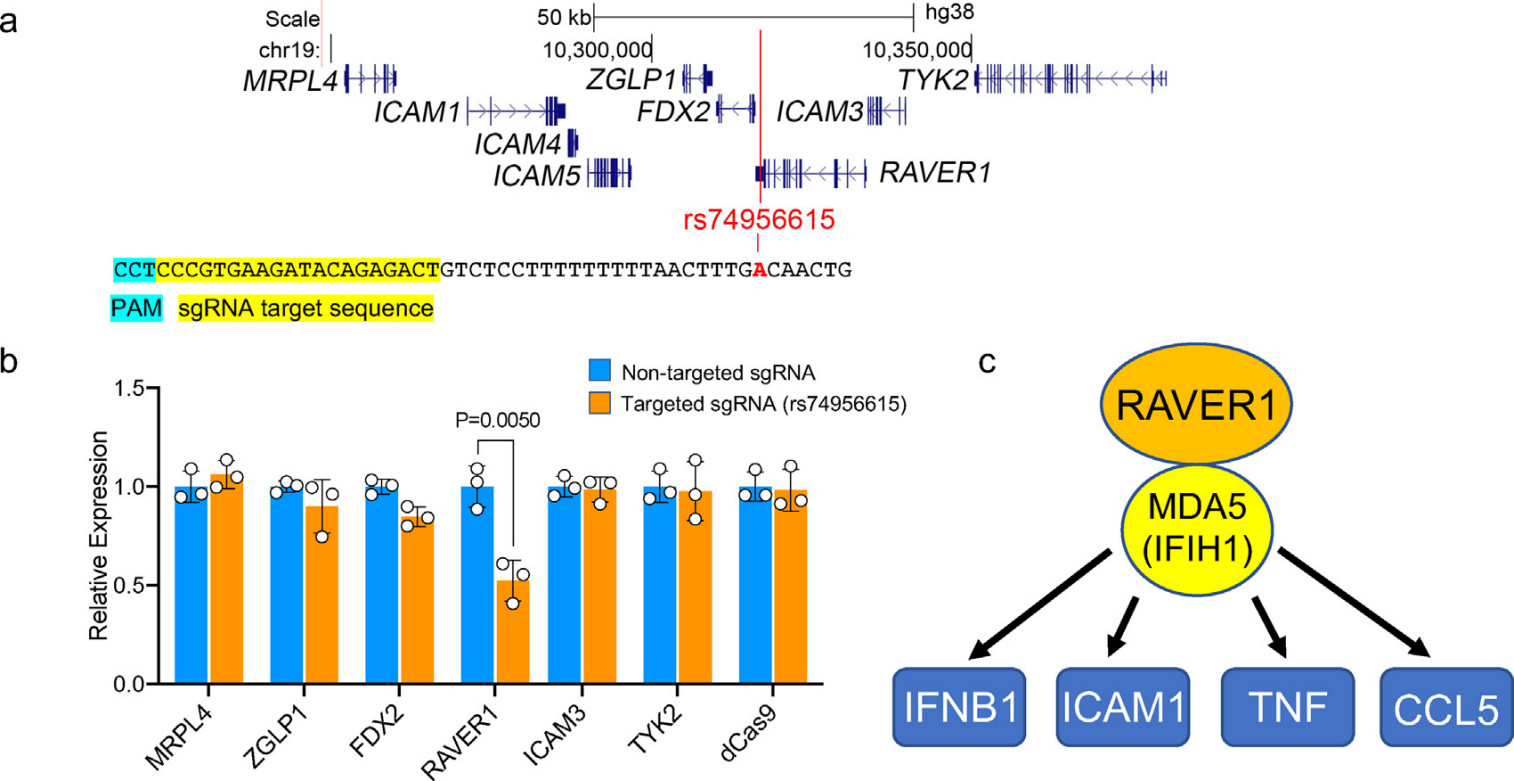
**An untranslated exonic region located near SNP rs74956615 that is linked to critical illness in COVID-19 regulates the expression of *RAVER1*.** (a) SNP rs74956615 (red), which is significantly associated with critical illness in COVID-19 is located at the 3’ untranslated exonic region (UTR) of *RAVER1* on chromosome 19p13.2. A gRNA target sequence (yellow highlighted) along with PAM sequence (blue highlighted) near rs74956615 that was used for subsequent CRISPRi analysis is shown. The untranslated exon sequence is in uppercase. (b) CRISPRi analysis targeting the 3’ UTR of *RAVER1* near rs74956615 was conducted using sgRNA targeting the 3’ UTR region in an A549 cell line that stably expresses dCas9-KRAB. The analysis indicates that the sgRNA repressed the expression of *RAVER1*, but no other expressed lung epithelial genes located nearby at chromosome 19p13.2 compared to non-targeted sgRNA. Three independent experiments were conducted. Error bars are ± SD. P values (two-sided Student's *t*-test) are reported for 1.5 or more-fold change in endogenous genes. (c) Schematic showing MDA5 (IFIH1)-RAVER1 interaction driving expression of antiviral genes IFNB1, ICAM1, TNF and CCL5.^35^

Additional SNPs that are associated with critical illness in COVID-19 have been identified by GWAS.^11^^,^^12^ As described above, we performed the CRISPRi analysis in dCas9-KRAB-expressing A549 cells using synthetic sgRNA targeting each locus, including rs74956615 (at the 3’ untranslated exonic region of *RAVER1*; Fig. 2a and Supplementary Fig. S2), rs2109069 (at intron 3 of *DPP9*; Supplementary Fig. S3), rs6489867 (at intron 5 of *OAS1*; Supplementary Fig. S4), rs10735079 (at intron 2 of *OAS3*; Supplementary Fig. S4) and rs2236757 (at intron 6 of *IFNAR2*; Supplementary Fig. S5). Notably, an sgRNA targeting a locus (yellow highlighted) near rs74956615 significantly repressed the expression of *RAVER1* but no other lung epithelial genes expressed in A549 cells, including *MRPL4, ZGLP1, FDX2, ICAM3* and *TYK*, located at chromosome 19p13.2 (≥1.5-fold, P=0.0050, two-sided Student's *t*-test; Fig. 2b), suggesting that the locus with rs74956615 influences the expression of *RAVER1* and in turn impacts the development of critical illness in COVID-19. RAVER1 is a co-activator of MDA5 (IFIH1), which recognizes nucleic acids associated with viral infections such as dsRNAs, including SARS-CoV-2, and activates antiviral response genes, including *IFNB1, ICAM1, TNF* and *CCL5* (Fig. 2c),^35^ suggesting that the MDA5 (IFIH1) antiviral pathway may be modified in patients carrying rs74956615 risk allele, which results in critical illness. The sgRNAs targeting other loci, including rs2109069, rs6489867, rs10735079 and rs2236757, did not affect the expression of nearby genes 1.5 or more-fold (Supplementary Figs. S3-S5).

### *ICAM1*, a downstream gene of the MDA5/RAVER1 pathway, is reduced in alveolar lung epithelial cells of COVID-19 patients

**Figure 3:**
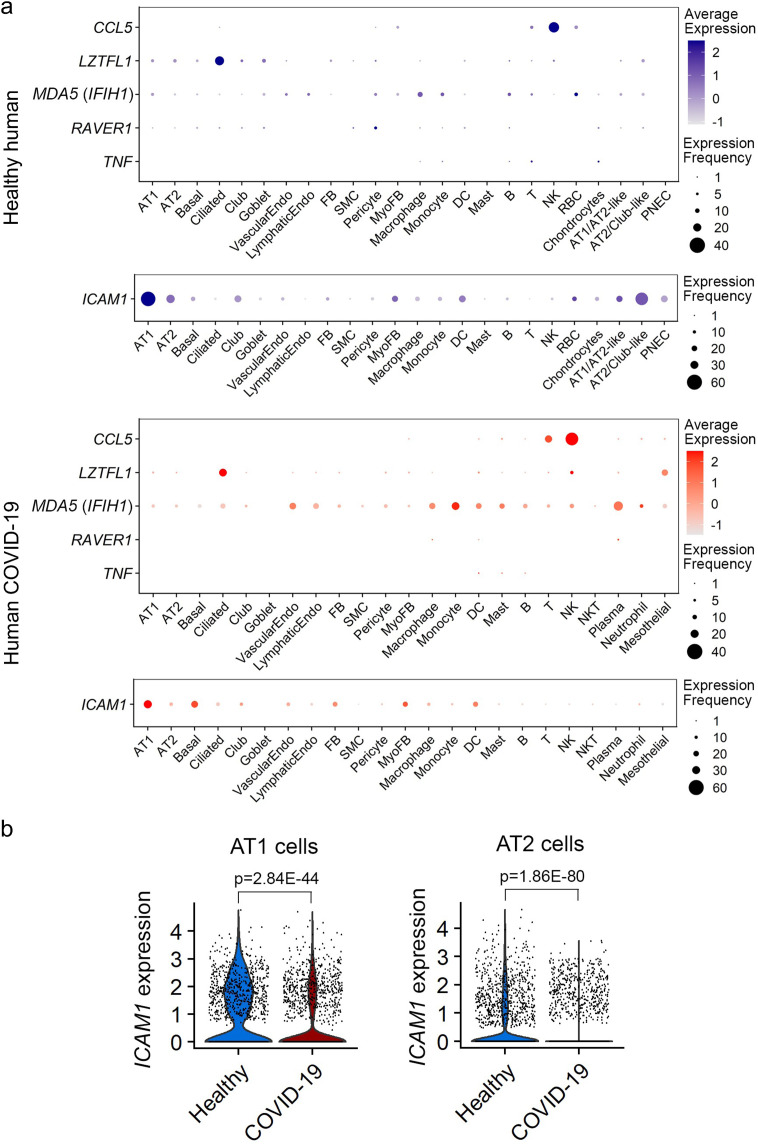
***ICAM1*, a downstream gene of the MDA5/RAVER1 pathway, is downregulated in alveolar AT1 and AT2 epithelial cells of COVID-19-succumbed human lungs compared to healthy lungs.** (a) Shown are dot plots obtained by snRNA-seq analysis that demonstrate the expression of *LZTFL1* and the MDA5 pathway-related genes (*RAVER1, IFIH1, ICAM1, TNF* and *CCL5*) in different cell types from lungs of healthy (top two panels; n=3) and COVID-19 succumbed (bottom two panels; n=5) humans (age<60). Dot colour and size indicate the average gene expression and percentage of cells expressing the gene, respectively. Gene expression in pDC cells was not visualized as there is only one predicted pDC cell in the COVID-19 data. (b) Shown are violin plots obtained by snRNA-seq analysis that demonstrate the relative expression level of ICAM1 in AT1 cells (left panel) and AT2 cells (right panel) of lungs of healthy (n=3) and COVID-19-succumbed (n=5) humans (age<60). P values were obtained using two-tailed Wilcoxon rank sum test. AT1: alveolar type I cells; AT2: alveolar type II cells; PNEC: pulmonary neuroendocrine cells; VascularEndo: vascular endothelial cells; LymphaticEndo: lymphatic endothelial cells; FB: fibroblasts; MyoFB: myofibroblasts; SMC: smooth muscle cells; DC: dendritic cells; pDC: plasmacytoid dendritic cells; NK: natural killer cells; NKT: natural killer T cells.

In order to understand the importance of LZTFL1 and the MDA5/RAVER1 antiviral pathway in COVID-19 disease severity influenced by the COVID-19 GWAS loci, the expression of *LZTFL1* and the MDA5/RAVER1 pathway-related genes were examined *in vivo* at a single cell level in COVID-19-infected lungs obtained from 5 donors deceased by COVID-19 (age<60) using recently reported snRNA-seq datasets.^22^^,^^23^ LZTFL1 was expressed in airway ciliated cells in both healthy and COVID-19-succumbed lungs (Fig. 3a, Supplementary Table S1), which is consistent with the previous report^34^^,^^36^ and the Human Protein Atlas data (https://www.proteinatlas.org/ENSG00000163818-LZTFL1/celltype) from healthy lungs, suggesting that the expression level of LZTFL1, which is affected by the COVID-19 GWAS locus (rs11385942 risk allele), can be critical for airway viral clearance. Notably, among the MDA5/RAVER1 pathway-related genes, *ICAM1* (but not *IFNB1, TNF* and *CCL5*), which is a downstream gene of MDA5 (IFIH1)/RAVER1,^35^ was significantly reduced in alveolar AT1/AT2 lung epithelial cells of COVID-19-succumbed human lungs compared to healthy lungs (Fig. 3a and b, Supplementary Table S1), the data of which was consistent with additional analysis of another snRNA-seq dataset obtained from COVID-19-succumbed lungs,^26^ albeit lower fold probably due to age ≥58 (up to 84) population dataset (Supplementary Table S2). ICAM1 (Intercellular adhesion molecule-1) in alveolar lung epithelial cells enhances adherence of neutrophils to alveolar lung epithelial cells upon lung inflammation,^37^ suggesting that the antiviral MDA5/RAVER1 pathway driving ICAM1-mediated lung neutrophil/epithelial interaction is critical in severely affected COVID-19 lungs.

### *ICAM1*, a downstream gene of MDA5/RAVER1 pathway, is induced in alveolar AT2 lung epithelial cells of COVID-19-resistant African green monkey lungs

**Figure 4:**
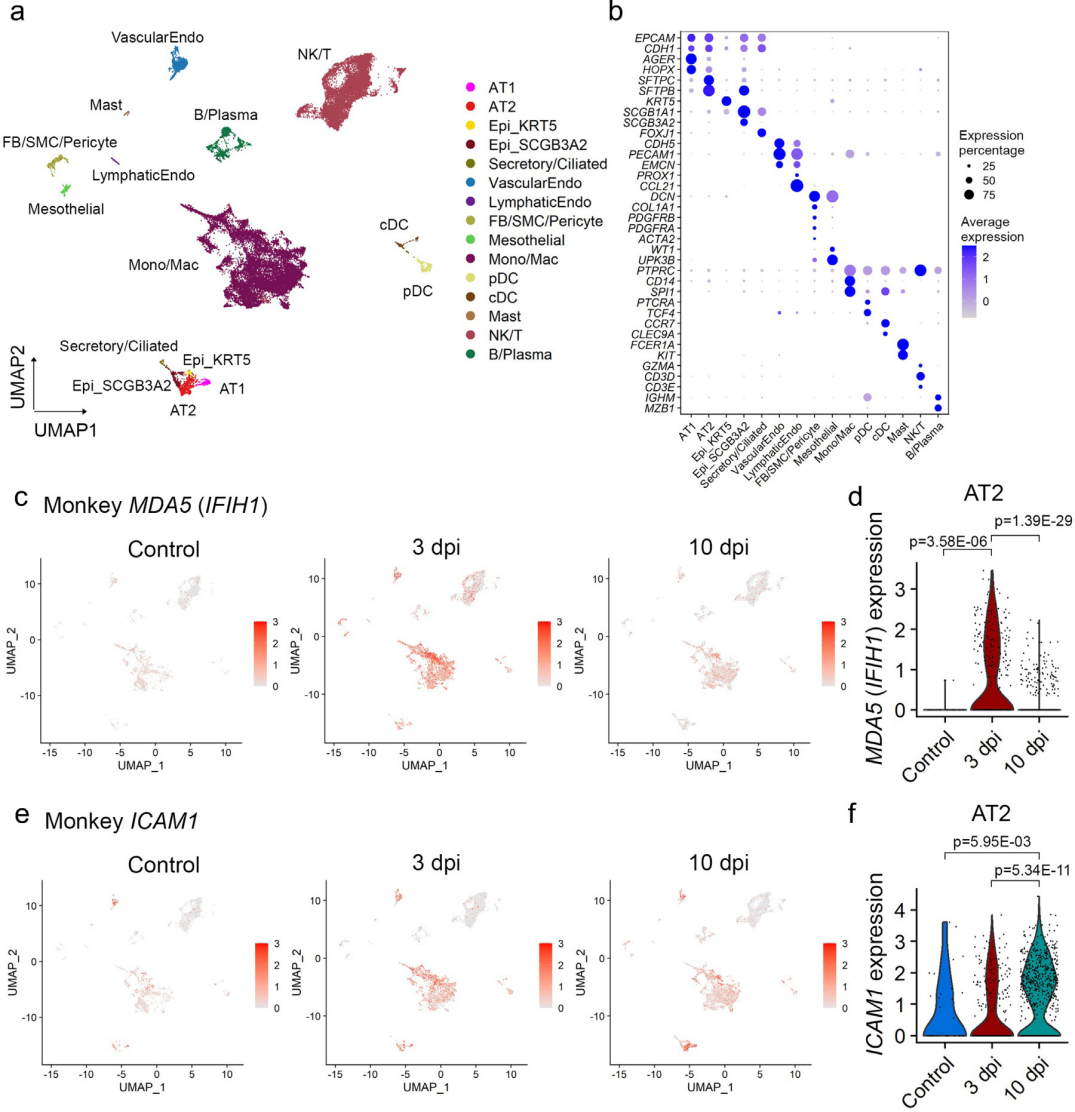
***MDA5 (IFIH1)* and *ICAM1* are induced in alveolar AT2 lung epithelial cells in African green monkeys that are experimentally infected with SARS-CoV-2.** (a) Shown is uniform manifold approximation and projection (UMAP) obtained by scRNA-seq analysis that demonstrates different cell populations existing in the lungs of African green monkeys infected with SARS-CoV-2 (n=2, 3 dpi of irradiated virus; n=4, 3 dpi of live virus; n=4, 10 dpi of live virus). (b) Shown are dot plots obtained by scRNA-seq analysis demonstrating that each cell population is defined by specific cell type gene markers. (c) Shown are UMAPs indicating the expression of *MDA5* (*IFIH1*) in SARS-CoV-2 infected monkey lungs at 3- and 10-days post infection (control, 3 dpi irradiated virus). (d) Violin plots indicate that *MDA5* (*IFIH1*) is induced at 3 dpi live SARS-CoV-2 compared to control in alveolar AT2 lung epithelial cells. (e) Shown are UMAPs indicating the expression of *ICAM1* in SARS-CoV-2 infected monkey lungs as described in (c). (f) Violin plots indicate that *ICAM1* is induced at 10 dpi compared to 3 dpi in alveolar AT2 lung epithelial cells. P values were obtained using two-tailed Wilcoxon rank sum test and adjusted using Bonferroni correction for multiple testing among the three groups. AT1: alveolar type I cells; AT2: alveolar type II cells; Epi_KRT5: epithelial cell cluster with selective expression of *KRT5*; Epi_SCGB3A2: epithelial cell cluster with selective expression of *SCGB3A2*; Secretory/Ciliated: secretory or ciliated cells; VascularEndo: vascular endothelial cells; LymphaticEndo: lymphatic endothelial cells; FB/SMC/Pericyte: fibroblast, smooth muscle, or pericyte cells; Mono/Mac: monocyte or macrophage cells; pDC: plasmacytoid dendritic cells; cDC: classical dendritic cells; NK/T: natural killer or T cells; B/Plasma: B or plasma cells.

To further assess the protective role of ICAM1 in lung epithelial cells against COVID-19 disease severity, we employed single-cell RNA-seq (scRNA-seq) datasets from lungs of an African green monkey COVID-19 disease model that was experimentally infected with SARS-CoV-2.^28^ Of note, African green monkeys develop only mild to moderate COVID-19 disease but not severe disease,^28^ suggesting that antiviral responses to SARS-CoV-2 are critical for the monkeys to survive. The monkey datasets provide scRNA-seq data of lungs from monkeys infected with irradiated (3 days post infection [3 dpi]; control) and live SARS-CoV-2 (3 dpi or 10 dpi).^28^ Using unbiased clustering analysis, we were able to separate lung epithelial populations, including AT1 and AT2 cells, from the monkey lungs infected with SARS-CoV-2 (Fig. 4a, Supplementary Fig. S6 and Table S3). Representative gene markers that define distinct cell populations are shown in Fig. 4b. Notably, differential gene expression analysis indicated that *MDA5 (IFIH1*) was significantly induced 3 days after SARS-CoV-2 experimental infection (3 dpi; Figs. 4c and 4d, Supplementary Fig. S7 and Table S3) and *ICAM1* was significantly induced 10 days after the infection in alveolar AT2 lung epithelial cells (10 dpi; Fig. 4e and f, Supplementary Fig. S7 and Table S3), suggesting that the antiviral MDA5/RAVER-ICAM1 pathway is activated in alveolar AT2 lung epithelial cells of SARS-CoV-2-resistant monkeys.

## Discussion

Since disparity in disease severity in COVID-19 patients cannot be explained just by age, sex and existing health conditions (e.g., a portion of young healthy persons regardless of sex die of COVID-19), a number of GWAS studies have been conducted using genomic DNA from COVID-19 patients, which resulted in the identification of chromosomal loci that are significantly associated with critical illness in COVID-19.^8^, ^9^, ^10^, ^11^, ^12^ However, since most of these loci are located at non-coding regions, it has been difficult to determine which neighbouring genes are affected by the SNP-containing loci. The expression of genes which are affected by these loci needs to be identified to develop therapeutic approaches that can target corresponding genes using drugs. In the present study, using CRISPRi and sn/scRNA-seq analyses, we identified *LZTFL1* and *RAVER1* that are expressed in airway ciliated cells and alveolar AT2 lung epithelial cells, respectively, as genes whose expression is affected by the loci that were identified by GWAS to be associated with COVID-19 severity.

In our study, we used lung epithelial cell lines since they are the first line of defence against the SARS-CoV-2 virus and respond to the virus physically and biologically using antiviral mechanisms.^32^ However, blood cells also play important roles in response to the virus especially when lung epithelial cells are not sufficient for eradication. In order to understand the role of the COVID-19 GWAS locus in blood cells that carry multiple variants, including SNP rs11385942, on *LZTFL1* at chromosome locus 3p21.21, Yao et al. deleted this portion of *LZTFL1* using a genome editor CRISPR/Cas9 in blood cell lines and found that the expression of *CCR9* and *SLC6A20* was affected by the locus,^38^ suggesting that this COVID-19 GWAS locus at chromosome locus 3p21.21 may influence different genes depending on cell type. Other COVID-19 GWAS loci, which did not affect the expression of neighbouring genes according to our present analysis using lung epithelial cell lines, may influence their expression in non-lung epithelial cells, including blood cells. In order to precisely assess the roles of COVID-19-associated SNPs, the creation of different types (e.g., epithelia or blood) of cell lines and/or the culture of primary cells that carry non-risk or risk alleles is required, which allows assessment of whether the expression of COVID-19-relevant genes are affected by risk alleles in different cell types even in the presence or absence of SARS-CoV-2. Of note, we designed our sgRNA sequences independently of conventional histone gene-regulatory marks and ATAC accessibility considering our previous experience that a portion of transcription factors can access ATAC-inaccessible regions,^19^ suggesting that assessing GWAS loci in an agnostic approach may lead to the identification of previously unrecognized gene-regulatory regions.

**Figure 5:**
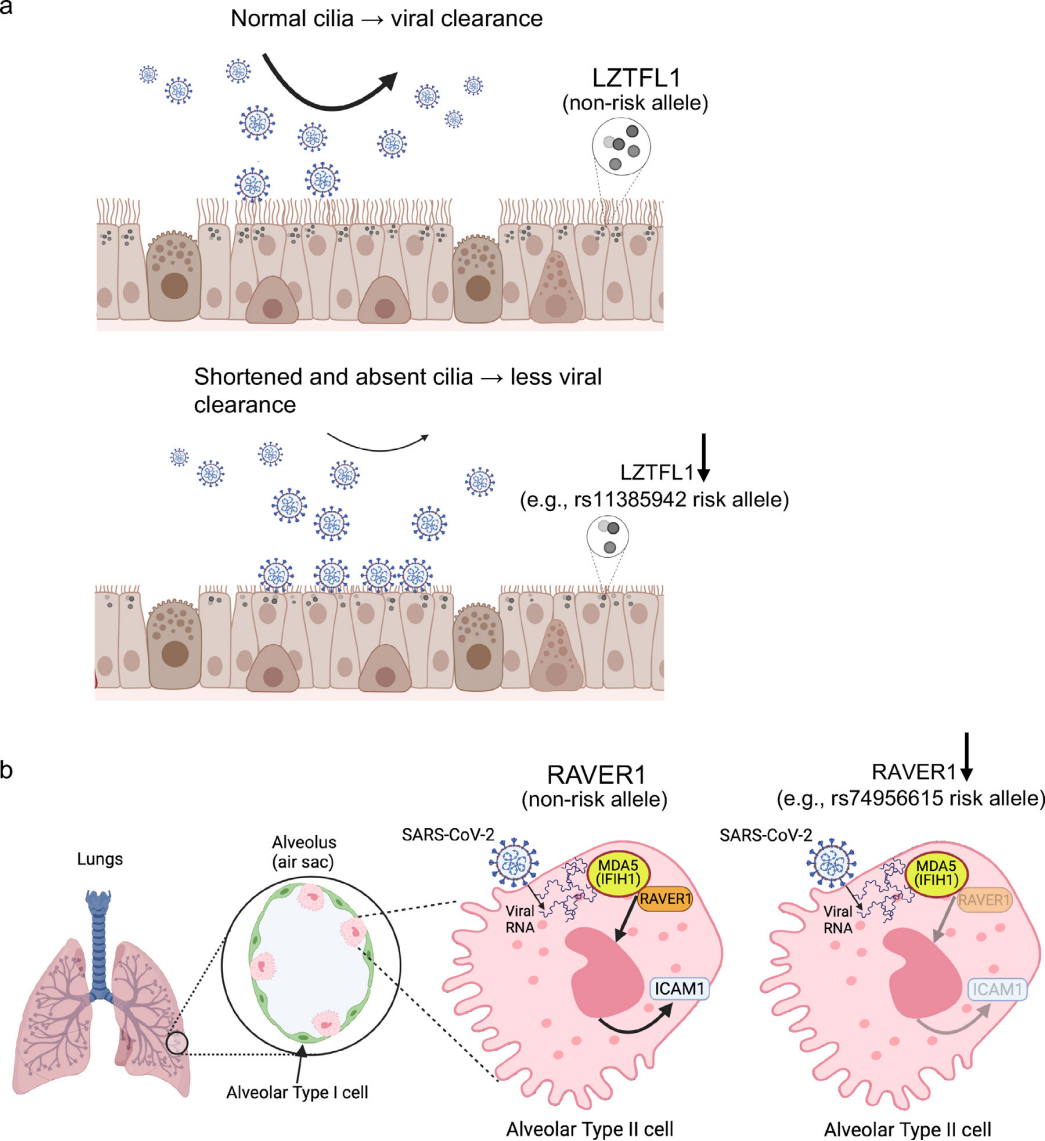
**Proposed models of the mechanisms by which COVID-19 GWAS loci influence COVID-19 disease severity.** (a) Human airway ciliated cells with intact LZTFL1 (non-risk allele) clear SARS-CoV-2 by efficient ciliary beating (top panel) while those with insufficient LZTFL1 (e.g., rs11385942 risk allele) cannot clear SARS-CoV-2 due to inefficient ciliary beating (bottom panel), thereby allowing more SARS-CoV-2 to enter alveolar lung regions where gas exchange occurs and in turn causing lung dysfunction. (b) In human alveolar AT2 lung epithelial cells that are infected with SARS-CoV-2, MDA5 (IFIH1) associated with its coactivator RAVER1 senses double-stranded RNA produced by SARS-CoV-2 and induces ICAM1 that recruits neutrophils to AT2 cells to combat SARS-CoV-2. In human AT2 cells that lack the proper antiviral mechanism mediated by MDA5 (IFIH1) due to insufficient RAVER1 (e.g., rs74956615 risk allele), ICAM1 may not be induced properly upon SARS-CoV-2 infection, thereby allowing the accumulation of SARS-CoV-2 in lung alveolar regions lacking sufficient neutrophils, causing excessive lung damage. The figure drawings were created with BioRender.com.

Our present study analysing COVID-19 GWAS loci led to the identification of *LZTFL1* and *RAVER1* as influencing COVID-19 disease severity. As our sn/scRNA-seq data and the data from others indicate, LZTFL1 is highly expressed in ciliated cells, including airway ciliated cells, and regulates ciliogenesis and ciliary function.^34^^,^^36^^,^^39^ Since the reduced expression of LZTFL1 leads to fewer airway ciliated cells with shorter cilia,^34^ patients who carry SNP rs11385942 risk allele may lose LZTFL1 expression rapidly upon SARS-CoV-2 virus infection. This can result in inefficient airway viral clearance by weakened cilia and in turn spur the accumulation of more SARS-CoV-2 virus in the lung leading to severe COVID-19 disease (Fig. 5a). Likewise, ageing has been shown to weaken airway ciliary function that leads to more frequent respiratory infection,^40^ which may also explain increased critical COVID-19 cases in elderly populations. Our analyses of sn/scRNA-seq datasets also indicate that *RAVER1* along with its interacting partner *MDA5* (*IFIH1*) and downstream gene *ICAM1* are expressed in lung epithelial cells, including alveolar AT2 cells, which is consistent with the Human Protein Atlas data using healthy lungs (https://www.proteinatlas.org/ENSG00000161847-RAVER1/celltype). RAVER1 functions as a co-activator for MDA5 (IFIH1)-mediated cellular antiviral responses, in part through neutrophil activation mediated by ICAM1 that is downstream of MDA5/RAVER1.^35^^,^^37^^,^^41^ The relatively low expression of RAVER1 in *in vivo* snRNA-seq datasets compared to the cell lines (https://www.proteinatlas.org/ENSG00000161847-RAVER1/celltype) might be due to the difficulty of obtaining RAVER1-positive cells through the sn/scRNA-seq approach akin to the reported dearth of neutrophils in the sn/scRNA-seq analysis.^25^ Patients who carry SNP rs74956615 risk allele may have reduced RAVER1 upon SARS-CoV-2 virus infection and fail to properly activate MDA5-mediated expression of antiviral genes, especially *ICAM1*, in alveolar AT2 lung epithelial cells. Then, patients with SNP rs74956615 risk allele may accumulate SARS-CoV-2 virus in the lung, especially in the alveolar region, which is important for gas exchange, leading to COVID-19 critical illness (Fig. 5b). As SARS-CoV-2 antagonizes MDA5 (IFIH1) activation to evade host immunity, MDA5 (IFIH1) has been shown to be critical for antiviral responses against SARS-CoV-2.^42^ Notably, children, who are more resistant to SARS-CoV-2 than adults, have elevated MDA5 (IFIH) pathway genes in the upper airways along with activated neutrophils compared to adults, who are more susceptible to SARS-CoV-2.^43^ This finding is consistent with our scRNA-seq analyses showing elevated expression of *MDA5* (*IFIH1*) and downstream *ICAM1* using the SARS-CoV-2-infected lungs of monkeys that are resistant to SARS-CoV-2 (Fig. 4). People who harbour risk alleles of SNPs rs11385942 and/or rs74956615 may benefit from early detection of SARS-CoV-2 virus by frequent PCR testing. If positive, immediate treatment using an antibody targeting SARS-CoV-2 (e.g., REGN-COV2)^44^ could be beneficial in reducing the level of SARS-CoV-2 virus as soon as possible considering that such people may have weakened ciliary function and/or impaired antiviral pathways, respectively, as indicated by the presence of risk alleles of the SNPs. However, in order to implement such a therapeutic strategy, further *in vitro* and *in vivo* studies to delineate the mechanisms will be required, especially considering that a small sample size was used for the present snRNA-seq analysis. Moreover, such diagnosis and associated therapy should be considered after assessing, in a prospective clinical trial, whether a single variant or a polygenic risk score (PRS) is more appropriate as a biomarker based on reliability and feasibility.^45^^,^^46^

Our present study using CRISPRi and sn/scRNA-seq datasets identifies possible genes that are functionally affected by the loci determined by GWAS to be associated with COVID-19 disease severity. Our data so far suggest that people who have risk alleles of SNPs rs11385942 and/or rs74956615 may carry impaired antiviral defence systems against SARS-CoV2 virus, thereby accumulating more virus and in turn more severe COVID-19 symptoms. Early detection of the virus by frequent PCR testing and immediate virus targeting therapy for people who carry risk alleles of SNPs rs11385942 and/or rs74956615 may be beneficial.

## Contributors

YM conceived the experiments. IMF-B, WDS and YM performed the experiments. MG analysed sn/scRNA-seq data. WDS and MG created figures. IMF-B, WDS, JJB, MG and YM wrote the paper. All authors verified and discussed the data, read and approved the final version of the manuscript.

## Declaration of interests

The authors declare no competing interests.

## Appendix Supplementary materials

Image, application 1

Image, application 2

Image, application 3
