# Supplementary material for: CRISPRi links COVID-19 GWAS loci to LZTFL1 and RAVER1
Source: eBioMedicine. 2022 Jan 6;75:103806. doi: 10.1016/j.ebiom.2021.103806 (PMC8731227; doi:10.1016/j.ebiom.2021.103806)
Supplement: Supplementary file 1 [file mmc1.pdf]

Genetica
CELL LINE TESTING
a LabCorp brand
1440 York Court, Burlington, NC 27215 USA www.celllineauthentication.com (513) 985-9777

Account Information
Account Number: **34138690**
CINCINNATI CHILDREN'S HOSPITAL
Acct Ref 1:
Acct Ref 2:
Acct Ref 3:
CINCINNATI, OH 45229

Cell Line Evaluation

LabCorp Case #   CX4-012259

Sample	Label/Reference	LabCorp Id	Received
Cell Line Sample 1	**A549 (p85)**	1AL-9076-0	10/21/2021
Cell Line Reference	**A-549 [A549] (PMID 25877200)**	1AL-9077-0	10/21/2021

DNA Analysis

	D3S1358	D7S820	vWA	FGA	D8S1179	D21S11	D18S51	D5S818	D13S317	D16S539
SMP1	16	8, 11	14	23	13, 14	29	14, 17	11	11	11, 12
REF	16	8, 11	14	23	13, 14	29	14, 17	11	11	11, 12

DNA Analysis

	TH01	TPOX	CSF1PO	AMEL	Penta D	Penta E	Mouse
SMP1	8, 9.3	8, 11	10, 12	X, Y	9	7, 11	NA*
REF	8, 9.3	8, 11	10, 12	X, Y	9	7, 11	NA

Conclusion:
Percent Match (ANSI/ATCC ASN-0002-2011): 100.00%
Percentage of Identity: 100.00%

Based on testing results obtained from analyses of 15 autosomal short tandem repeat (STR) loci and the gender identity locus amelogenin, the profile of the submitted sample (1AL-9076-0), labeled A549 (p85), is a 100.00% match to the reference profile of A-549 [A549] (PMID 25877200). These data support the authentication of A549 (p85) as compared to the reference profile of A-549 [A549] (PMID 25877200). The electropherograms of analyzed data are attached. No mouse DNA detected for sample 1AL-9076-0.

Percent match is calculated by dividing the number of identical alleles by the total number of surveyed alleles in the questioned profile only. This calculation does not take into account loss of heterozygosity in the questioned profile (ANSI/ATCC ASN-0002-2011). Percentage of Identity is calculated by dividing twice the number of identical alleles by the total number of surveyed alleles in the questioned and reference profiles (Tiss. Cult. Res. Commun. 18:329, 1999). Please note that homozygous alleles count as one allele.

The source of the reference STR profile, whether generated in-house or obtained from an external source, is listed in parentheses next to the reference sample name.

*NA- Allele Information not available.

Megan D Shaffer, Ph.D.
Technical Director
DNA Identification Testing Division
Laboratory Corporation of America Holdings
October 25, 2021

Laboratory Corporation of America Holdings (LabCorp), 1440 York Court, Burlington, NC is accredited to ISO/IEC 17025:2005 by ANSI National Accreditation Board (ANAB), for STR analysis.

Genetica
CELL LINE TESTING
a LabCorp brand
1440 York Court, Burlington, NC 27215 USA www.celllineauthentication.com (513) 985-9777

Account Information
Account Number: **34138690**
CINCINNATI CHILDREN'S HOSPITAL
Acct Ref 1:
Acct Ref 2:
Acct Ref 3:
CINCINNATI, OH 45229

Cell Line Evaluation

LabCorp Case # CX4-012258

**Sample**	**Label/Reference**	**LabCorp Id**	**Received**
Cell Line Sample 1	**H1793 (p12)**	1AL-9074-0	10/21/2021
Cell Line Reference	**NCI-H1793 [H1793] (PMID 25877200)**	1AL-9075-0	10/21/2021

DNA Analysis

	D3S1358	D7S820	vWA	FGA	D8S1179	D21S11	D18S51	D5S818	D13S317	D16S539
SMP1	17, 18	7, 8	18	23.2, 24	8, 13	28	10, 18	11, 12	8, 12	13
REF	17, 18	7, 8	18	23.2, 24	8, 13	28	10, 18	11, 12	8, 12	13

DNA Analysis

	TH01	TPOX	CSF1PO	AMEL	Penta D	Penta E	Mouse
SMP1	9, 9.3	8	9, 12	X	13, 14	14, 16	NA*
REF	9, 9.3	8	9, 12	X	13, 14	14, 16	NA

Conclusion:

Percent Match (ANSI/ATCC ASN-0002-2011): 100.00%
Percentage of Identity: 100.00%

Based on testing results obtained from analyses of 15 autosomal short tandem repeat (STR) loci and the gender identity locus amelogenin, the profile of the submitted sample (1AL-9074-0), labeled H1793 (p12), is a 100.00% match to the reference profile of NCI-H1793 [H1793] (PMID 25877200). These data support the authentication of H1793 (p12) as compared to the reference profile of NCI-H1793 [H1793] (PMID 25877200). The electropherograms of analyzed data are attached. No mouse DNA detected for sample 1AL-9074-0.

Percent match is calculated by dividing the number of identical alleles by the total number of surveyed alleles in the questioned profile only. This calculation does not take into account loss of heterozygosity in the questioned profile (ANSI/ATCC ASN-0002-2011). Percentage of Identity is calculated by dividing twice the number of identical alleles by the total number of surveyed alleles in the questioned and reference profiles (Tiss. Cult. Res. Commun. 18:329, 1999). Please note that homozygous alleles count as one allele.

The source of the reference STR profile, whether generated in-house or obtained from an external source, is listed in parentheses next to the reference sample name.

*NA- Allele information not available.

Megan D Shaffer, Ph.D.
Technical Director
DNA Identification Testing Division
Laboratory Corporation of America Holdings
October 25, 2021

Laboratory Corporation of America Holdings (LabCorp), 1440 York Court, Burlington, NC is accredited to ISO/IEC 17025:2005 by ANSI National Accreditation Board (ANAB), for STR analysis.

PWD1
