# Supplementary material for: CRISPRi links COVID-19 GWAS loci to LZTFL1 and RAVER1
Source: eBioMedicine. 2022 Jan 6;75:103806. doi: 10.1016/j.ebiom.2021.103806 (PMC8731227; doi:10.1016/j.ebiom.2021.103806)
Supplement: Supplementary file 3 [file mmc3.pdf]

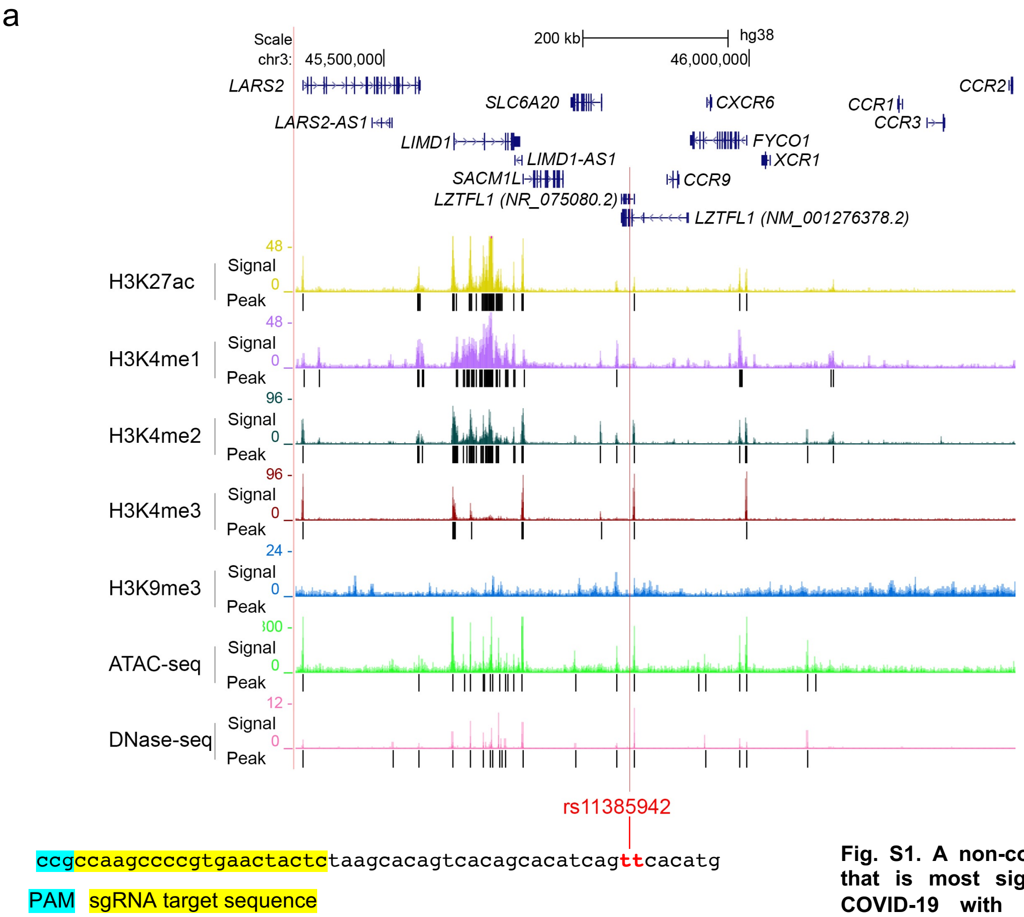

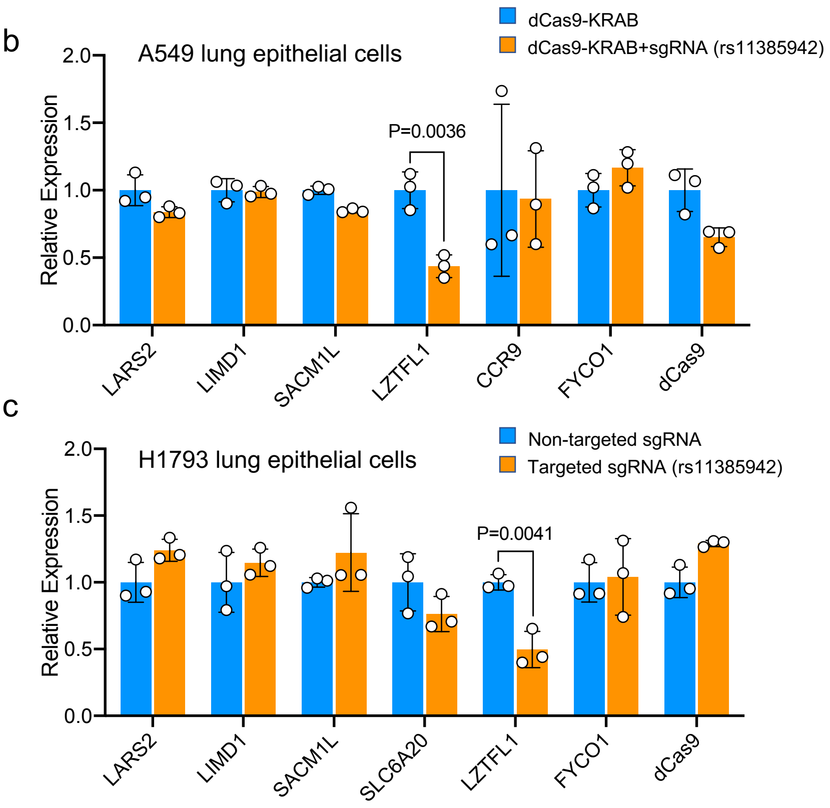

Fig. S1. A non-coding region near SNP rs11385942 that is most significantly associated with severe COVID-19 with respiratory failure regulates the expression of *LZTFL1* but not that of other nearby genes expressed in lung epithelial cell lines.

(a) SNP rs11385942 is located at intron 5 (or intron 3 for another isoform) of *LZTFL1* on chromosome 3p21.31. Rs11385942 is not associated with chromatin modifications in the A549 lung epithelial cell line shown by the ENCODE project. A guide RNA (gRNA) target sequence (yellow highlighted) along with PAM sequence (blue highlighted) near rs11385942 that was used for subsequent CRISPRi analysis is shown at the bottom.
(b) CRISPRi analysis was conducted using an A549 lung epithelial cell line that was infected with lentivirus harboring dCas9-KRAB and a single gRNA (sgRNA) targeting the intronic region near rs11385942. The analysis indicates that the sgRNA repressed the expression of *LZTFL1* but not that of other lung epithelial genes located near 3p21.31, which is consistent with the result using synthetic sgRNA described in Fig. 1b. Lentivirus that harbors only dCas9-KRAB was used as control.
(c) CRISPRi analysis that targets the intronic region near rs11385942 was conducted as described in Fig. 1b except that an H1793 lung epithelial cell line, which expresses endogenous *SLC6A20* and ectopic dCas9-KRAB, was used. The analysis indicates that the synthetic sgRNA repressed the expression of *LZTFL1* but not the other lung epithelial genes located nearby at 3p21.31 compared to non-targeted sgRNA, which is consistent with the result shown in Fig. 1b using A549 cells. Three independent experiments were conducted. Error bars are ± SD. P values (two-sided Student's *t*-test) are reported for 1.5 or more-fold change in endogenous genes.

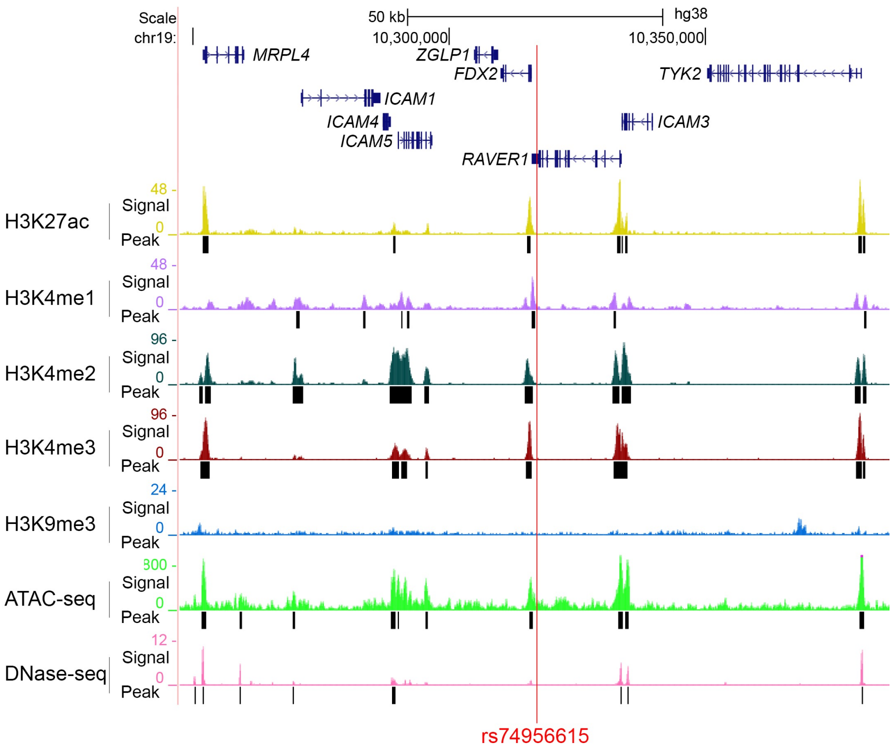

Fig. S2. A non-coding region near SNP rs74956615 that is linked to critical illness in COVID-19 at the 3' untranslated exonic region (UTR) of *RAVER1* on chromosome 19p13.2 is not associated with chromatin modifications.

Shown are chromatin modifications on chromosome 19p13.2 in an A549 lung epithelial cell line analyzed by the ENCODE project.

a

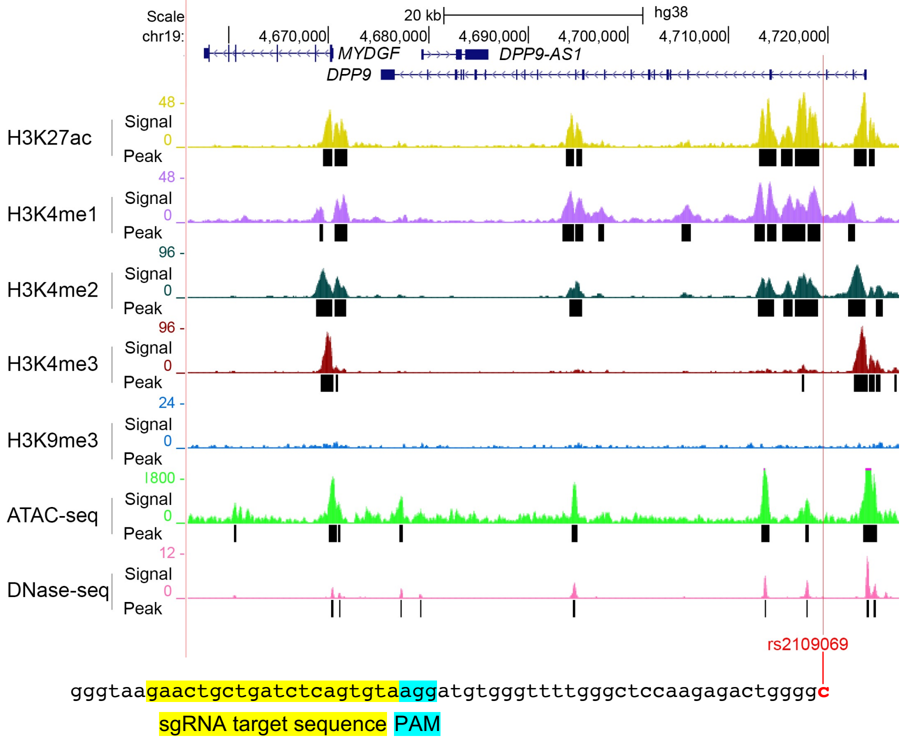

b

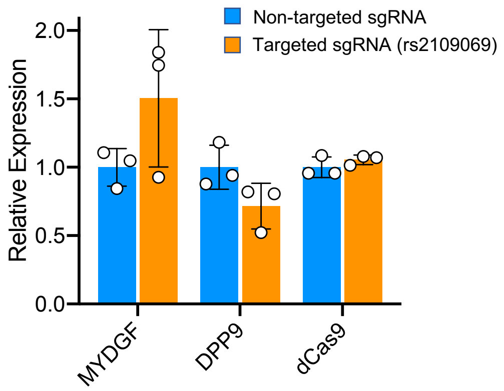

Fig. S3. A non-coding region near SNP rs2109069 that is significantly associated with critical illness in COVID-19 does not affect the expression of *DPP9* or *MYDGF* in an A549 lung epithelial cell line.

(a) SNP rs2109069 is located at intron 3 of *DPP9* on chromosome 19p13.3. Rs2109069 is not associated with chromatin modifications in an A549 lung epithelial cell line shown by the ENCODE project. A guide RNA (gRNA) target sequence (yellow highlighted) along with PAM sequence (blue highlighted) near rs2109069 that was used for subsequent CRISPRi analysis is shown at the bottom.
(b) CRISPRi analysis was conducted using synthetic single gRNA (sgRNA) targeting the intronic region near rs2109069 in an A549 lung epithelial cell line stably expressing dCas9-KRAB. The analysis indicates that the expression of genes (*DPP9* and *MYDGF*) near rs2109069 was not affected by the sgRNA compared to non-targeted control sgRNA. Three independent experiments were conducted. Error bars are ± SD. P values (two-sided Student's *t*-test) are reported for 1.5 or more-fold change in endogenous genes.

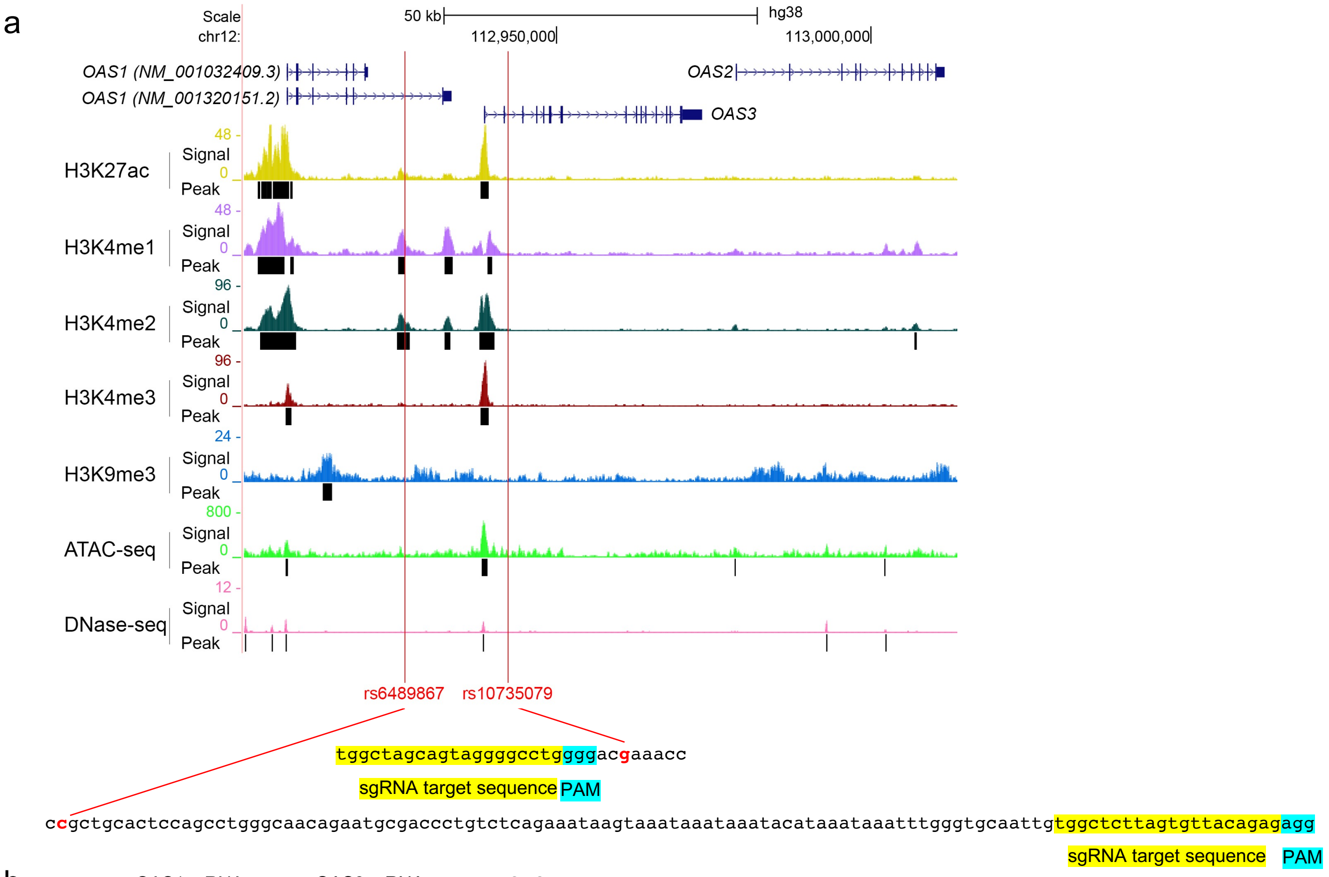

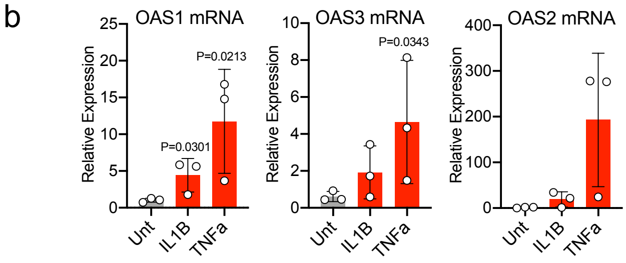

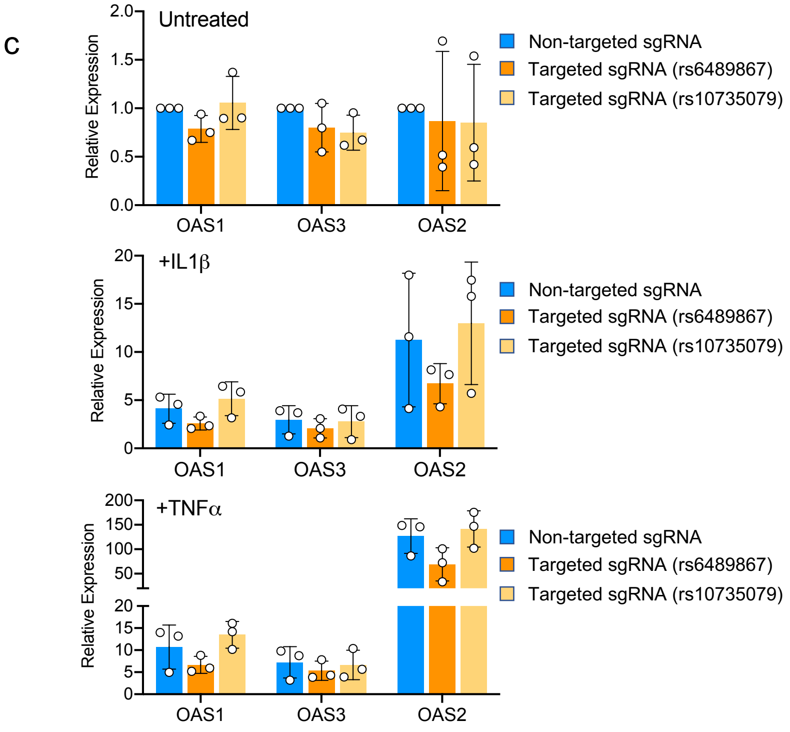

Fig. S4. Non-coding regions near SNPs rs6489867 and rs10735079 that are significantly associated with critical illness in COVID-19 do not affect the expression of *OAS1*, *OAS2* and *OAS3* in an A549 lung epithelial cell line.

(a) SNPs rs6489867 and rs10735079 are located at an intergenic region of *OAS1* and *OAS3* (or intron 5 of *OAS1*) and intron 2 of *OAS3* on chromosome 12q24.13, respectively. Rs6489867 but not rs10735079 is associated with chromatin modifications in an A549 lung epithelial cell line analyzed by the ENCODE project. The guide RNA (gRNA) target sequences (yellow highlighted) along with PAM sequences (blue highlighted) near rs6489867 and rs10735079 that were used for subsequent CRISPRi analysis are shown at the bottom.
(b) *OAS1*, *OAS3* and *OAS2* are induced by IL1β or TNFα in A549 lung epithelial cells. A549 cells were treated with IL1β or TNFα for 24 hours at the final concentration of 50 ng/ml and RNA was extracted for TaqMan gene expression analysis. All cells were transfected with non-targeted control sgRNA.
(c) CRISPRi analysis was conducted using synthetic single gRNAs (sgRNA) targeting the non-coding regions near rs6489867 and rs10735079 in an A549 lung epithelial cell line that stably expresses dCas9-KRAB in the presence/absence of IL1β or TNFα (50 ng/ml final concentration for both) for 24 hours that induced the expression of *OAS1*, *OAS3* and *OAS2*. The analysis indicates that the expression of genes (*OAS1*, *OAS2* and *OAS3*) near rs6489867 or rs10735079 was not affected by each sgRNA compared to non-targeted control sgRNA. Three independent experiments were conducted. Error bars are ± SD. P values (4b, ratio paired Student's *t*-test; 4c, two-sided Student's *t*-test) are reported for 1.5 or more-fold change in endogenous genes.

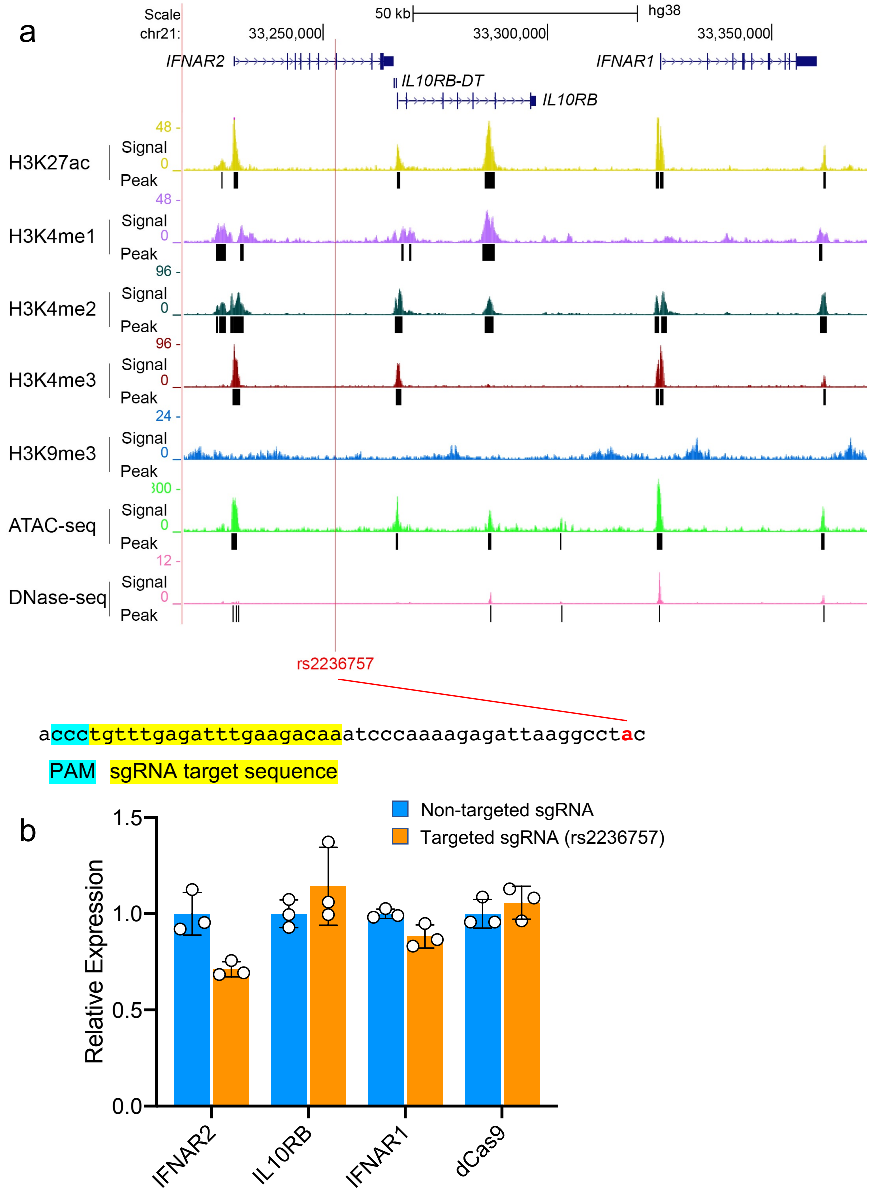

Fig. S5. A non-coding region near rs2236757 that is significantly associated with critical illness in COVID-19 does not affect the expression of *IFNAR2*, *IL10RB* or *IFNAR1* in an A549 lung epithelial cell line.

(a) SNP rs2236757 is located at intron 6 of *IFNAR2* on chromosome 21q22.1. Rs2236757 is not associated with chromatin modifications in an A549 lung epithelial cell line analyzed by the ENCODE project. A guide RNA (gRNA) target sequence (yellow highlighted) along with PAM sequence (blue highlighted) near rs2236757 that was used for subsequent CRISPRi analysis is shown at the bottom.
(b) CRISPRi analysis was conducted using synthetic single gRNA (sgRNA) targeting the intronic region near rs2236757 in an A549 lung epithelial cell line that stably expresses dCas9-KRAB. The analysis indicates that the expression of genes (*IFNAR2*, *IL10RB* or *IFNAR1*) near rs2236757 was not affected by the sgRNA compared to non-targeted control sgRNA. Three independent experiments were conducted. Error bars are ± SD. P values (two-sided Student's *t*-test) are reported for 1.5 or more-fold change in endogenous genes.

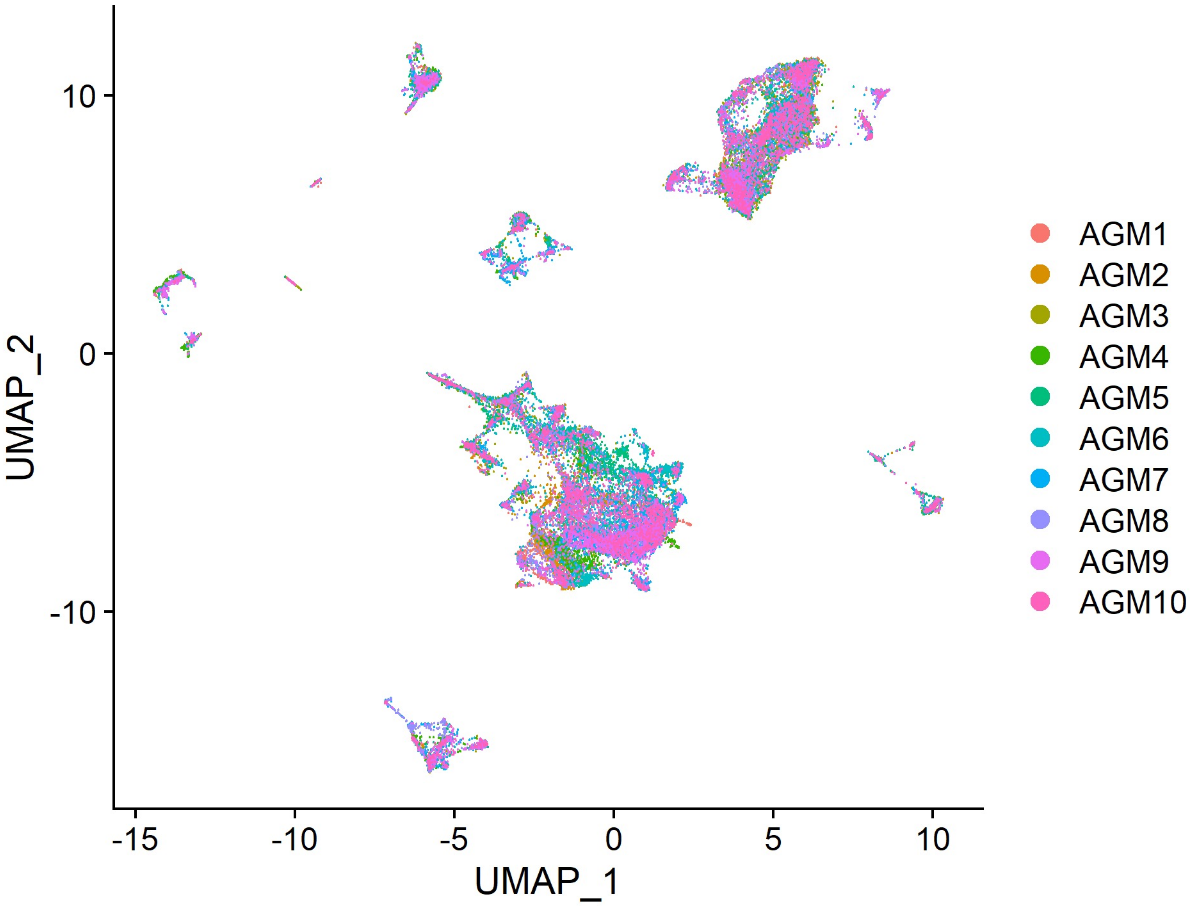

Fig. S6. Uniform manifold approximation and projection (UMAP) groups of different cell populations from lungs of African green monkeys infected with SARS-CoV-2.

The scRNA-seq data from each monkey lung at 3 days post infection (dpi) of irradiated (AGM1 and AGM2) or live (AGM3-AGM6) and 10 dpi of live (AGM7-AGM10) SARS-CoV-2.

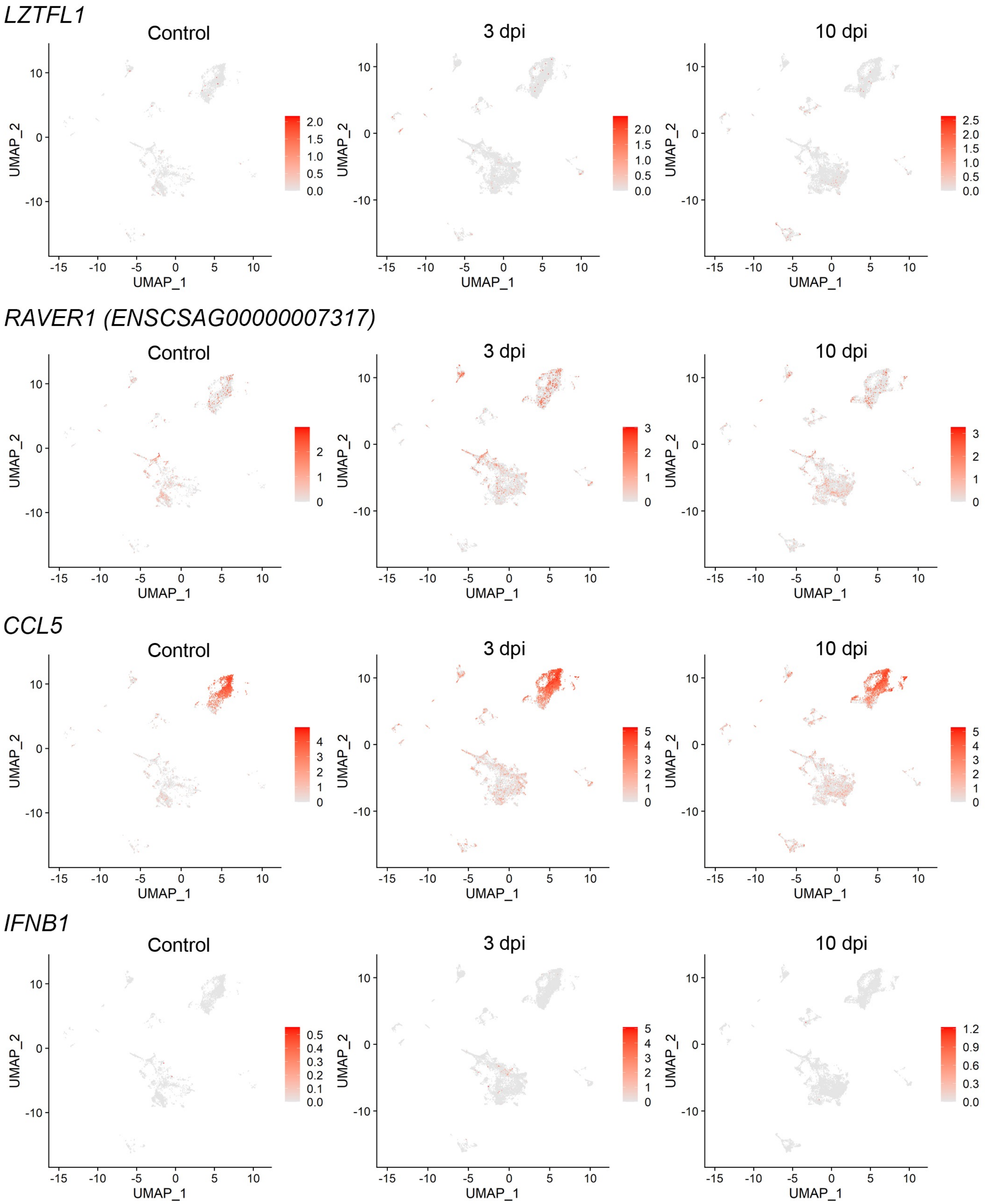

Fig. S7. The expression of *LZTFL1*, *RAVER1*, *CCL5* and *IFNB1* was not induced in lung epithelial cells of African green monkeys infected with SARS-CoV-2.

Shown are UMAPs indicating the expression of *LZTFL1*, *RAVER1*, *CCL5* and *IFNB1* (top to bottom panels) in SARS-CoV-2 infected monkey lungs (3 dpi or 10 dpi; control, 3 dpi irradiated virus). ENSCSAG00000007317 is equivalent to RAVER1 in African green monkey (Vervet-AGM, also known as *Chlorocebus sabaeus*).
